# Open elbow dislocation associated with distal ischemia in children about one case and review of the literature

**DOI:** 10.11604/pamj.2015.21.128.6553

**Published:** 2015-06-16

**Authors:** Noureddine Redjil, Mwinyanne Narcisse Dabire, Pierre Weber

**Affiliations:** 1 Service d'Orthopédie et de Traumatologie, Centre Hospitalier de Mulhouse, Mulhouse, France

**Keywords:** Children, ischimia, elbow dislocation, open, treatment

## Abstract

Elbow's dislocations of the child are rare injuries. Its associated injuries such as the opening and the distal ischemia are an extreme therapeutic emergency. One case of open elbow dislocation with distal ischemia in a 10-year-old child is reported. In the clinical examination, there is a deformity of the elbow, a wound showing the cartilaginous distal humerus with the brachial artery under tension on the trochlea that creates a beginning distal ischemia and hypoesthesia in the territory of the median nerve. The surgical health care included a careful debridement, a reduction and scanning of the neurovascular bundle of humerus with reappearance of pulses after 45 minutes. After the last follow-up at three months, the child's examination does not show any neurovascular disorder with a steady elbow. The functional prognosis depends mainly on the reduction time, the importance of neurovascular injuries and the skin opening.

## Introduction

The intermediate joint of the upper limb, the elbow is a set of complex joint including the distal extremity of the humerus and the proximal extremity of radius and ulna. The elbow dislocation is the most frequent child and adolescent dislocations. It represents 3% of elbow injuries in that population, and 3-6% of all elbow's injuries with a peak prevalence between 3 and 14 years old, the posterior dislocation is the most frequent. These dislocations are often complex, and can be associated to a major injury of medial capsular ligaments plane, to fractures or mostly to neurovascular injuries, the last ones are mainly observed when there are displaced supracondylar fractures. For the effectives dislocations, the vascular complication by brachial artery injury is more serious, its prevalence is estimated between 0.3 and 1.7% [1], it may be secondary to injury with severe valgus causing stretching of brachial artery and its rupture, it is the most common in open dislocations, the medical treatment will essentially depend on the nature of the vascular lesion, allowing mainly to determine the origin of ischemia and its reversibility or not, namely a stretching, vascular contusion or arterial spasm whose the prognosis is usually favorable, an effective vascular injury such as laceration with functional prognosis and life threatening limb's complication. One case of elbow's open dislocation in an 10 years-old child with distal ischemia and neurological deficit is reported.

## Patient and observation

**Figure 1:**
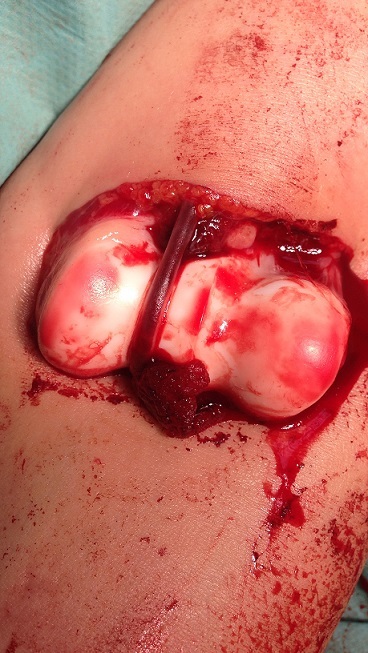
Open elbow dislocation with brachial artery stretched over the medial epicondyle

**Figure 2:**
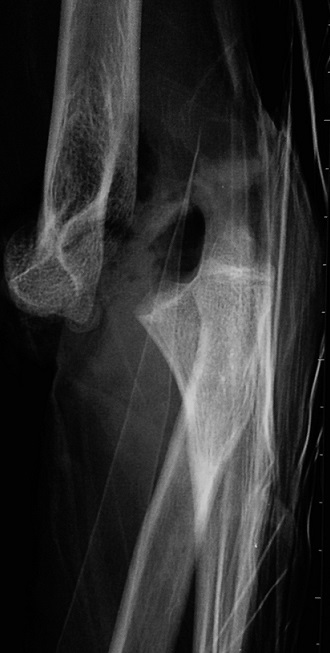
The lateral radiograph of the elbow dislocation

**Figure 3:**
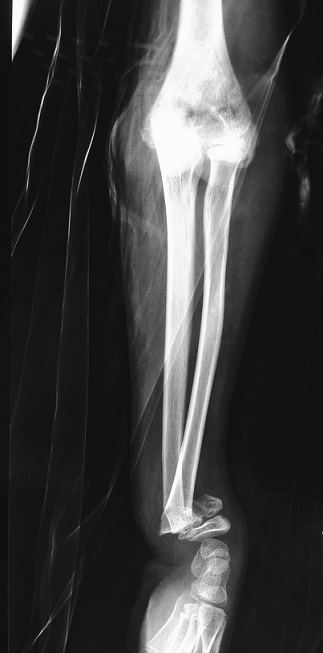
The wrist lateral radiograph showing epiphyseal separation fractures of the radius

**Figure 4:**
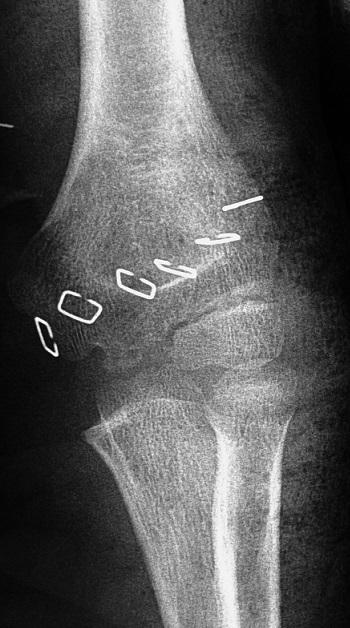
Elbow radiograph after reduction (face)

**Figure 5:**
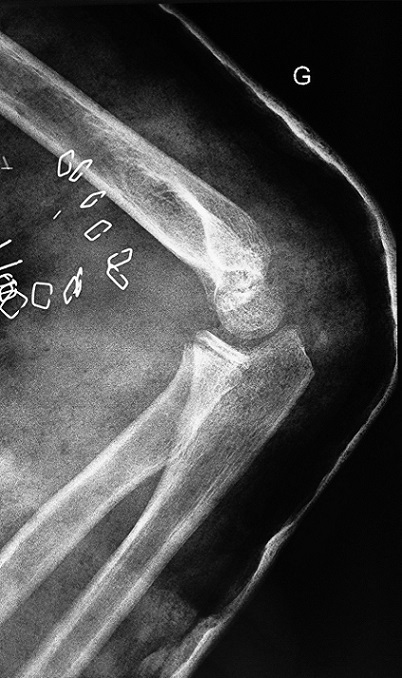
Elbow radiograph after reduction (profile)

**Figure 6:**
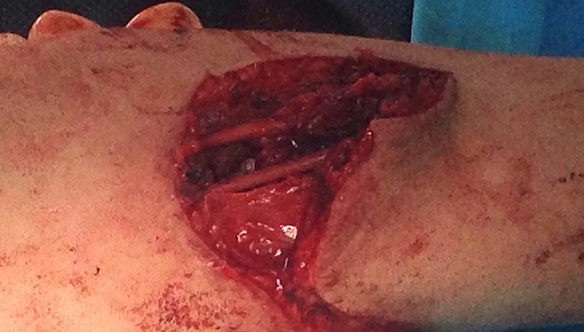
Muscle damage, the contused brachial artery and the median nerve after reduction of the dislocation

**Figure 7:**
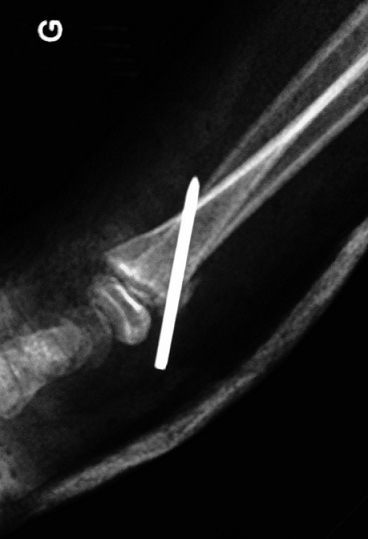
Radiograph of the wrist after reduction and pinning

It is 10 years-old child named BM, victim of a fall roughly 2 meters with reception on the left wrist in extension, causing an elbow's open injury and closed injury of ipsilateral wrist. The child was brought to the emergency department by his parents after a delay of 3 hours, without any pre-treatment. The entrance exam showed an functional disability of the left upper limb, a vicious attitude with an elbow deformation which looks like an ax, a horizontal contaminated wound approximatively 03 centimeters in the antecubital fossa, Cauchoix and Duparc type 2, exposing the cartilaginous distal humerus with a contused brachial artery under tension on the trochlear groove that creates a real easel (Figure 1), with the absence of pulsations on the visible portion of the brachial artery with abolition of distal pulses. However the hand remained hot and well colored. Furthermore the child had no motor deficit but a hypoesthesia in the territory of the median nerve. There was also a swelling with silverfork deformity of the wrist. The radiological evaluation revealed a convergent posterior elbow dislocation and epiphyseal fracture abruption (Figure 2), salter and Harris type 2 of the radius distal extremity (Figure 3). The surgery care, 30 minutes after admission, was first to a careful decontamination and debridement of the traumatic wound, followed by a reduction of the elbow's dislocation by External maneuvers (Figure 4, Figure 5). Showing the median nerve, which get back to its anatomical position and leading to the vanishing of the tension on the brachial artery (Figure 6). There is also a section at the myotendinous junction of brachialis. Firstly the radius distal extremity fracture was reduced, and then stabilized with a pin (Figure 7). In the presence of vascular surgeon with radial pulse's vanishing despite the reduction and with the warm and well colored hand, an exploration of the brachial artery was performed thirdly, the most likely lesion was vascular spasm on a bruised artery, it is what motivated us to apply papaverine and warm saline during 45 minutes, the necessary time needed to get the radial pulse back. The elbow's testing found an instability at 30^°^ of flexion, worsened by the rupture of the anterior musculo-capsular plane, a capsular plane's suture of brachialis mussel and wound closure were performed, the upper limb was immobilized in a brachiocephalic ante-brachial plaster splint. After 03 weeks, the child was seen by the doctor again and had no neurovascular deficit, the self-rehabilitation was started at the elbow while keeping the wrist immobilized in an antebrachial resin, the removal of the pin was performed after 06 weeks. After 03 months later, the mobility of the elbow and wrist is satisfactory.

## Discussion

Ranking as second in the adult after the dislocation of the shoulder, elbow dislocation is most common in children. It is due to significant trauma given the elbow's stability which is related to its bone configuration, its latero-internal ligaments (medial) and external (lateral). Associated lesions often are generated that significantly influence the functional prognostic of the joint, namely skin opening with infection's risk which is a serious complication, this risk depend on the degree of sepsis of the wound and the elapsed time since the accident, this skin opening is due to an excess initial movement and high energy trauma. Those occurred circumstances of dislocation must be an alert to look for an associated vascular injury, because of its evolving risk and it will be the first lesion to search during the initial clinical examination of injured limb in order to shorten the ischemia time and avoid the most serious complication which is the evolution towards Volkmann's syndrome, whose the occurrence is rare from 0.1 to 0.3% [2], but with heavy health effects [3]. The interest of health care treatment in a multidisciplinary center for this type of injury (trauma surgeon, vascular surgeon and radiologist) must be emphasized. The absence of pulse is not in itself a surgical emergency, unlike hypoperfusion signs [4], its cause might be either a simple compression or a more serious injury such as arterial section or intimal dissection. Once the dislocation is reduced, two situations are observed, first, the pulse, usually get back after a few minutes, reflecting an artery compression by displacement, but if the pulse does not get back, it reflects the artery is either spasmed or torn, it is in that case that the vascular exploration is still under debate [5].

Secondly, when there is no pulse associated with profound ischemia signs, brachial artery exploration is required, but with a warm and pink hand, the absence of the pulse led some teams to systematically realize an angiography in emergency [6], whereas in the other side, several reports have showed that angiography is a useless test and has no effect on treatment [7], we did not opt for this test which seems further more aggressive than a simple Doppler ultrasound. Finally when the arterial spasm that can be removed in 10 to 15 minutes by applying lidocaine and warm saline, is not sufficient to obtain good reperfusion, some authors choose immediately the vascular reconstruction [4], while Sabharwal et al, found that the early repair of the brachial artery is associated with a high rate of symptomatic re-occlusion and residual stenosis, and therefore a close observation period is recommended, with frequent neurovascular check-up before any more invasive Correction [7]. Given this complex debate, our health care treatment is based on the conservative attitude, after exploration of the artery which appeared bruised and application of papvérine and warm saline, and it took 45 minutes to get the radial pulse back. The most common neurological complication, associated with injuries of the elbow and especially during the displaced supracondylar fractures in children is neurapraxia. However, some reports of the impact vary widely, ranging from 2% to 35%. The damage of the anterior interosseous nerve is the most common and is reported in 21% of cases, the median nerve from 0% to 17% of cases [8]. Three factors seem to be taken into consideration before any indication of an early surgery, the existence of other associated complications, the type of paralysis and the nerve affected. The constant good prognosis of incomplete damaged nerve seems to make lawful the surgical abstention. In our case, the child has recovered normal sensitivity on the territory of the median nerve after three weeks. The broken wrist that has required a reduction by pinning for our patient did not rise too much debate about the therapeutic health care, unlike the medial epicondyle fracture which is associated with elbow's dislocation.

## Conclusion

The open dislocation of the elbow in children is rare, it is a serious injury with an uncertain prognosis, with associated injuries, bone, skin and neurological injuries which worsen more the situation, however only the presence of vascular injury with distal ischemia is dreadful, its health care treatment is still not codified, but should be multidisciplinary.

## References

[1] Ayel JE, Bonnevialle N, Lafosse JM, Pidhorz L, Al Homsy M, Mansat P, Chaufour X, Rongieres M, Bonnevialle P (2009). Acute elbow dislocation with arteriel rupture: analyse of nine cases. Orthop Traumatol Surg Res..

[2] Battaglia TC, Armstrong DG, Schwend RM (2002). Factors affecting forearm compartment pressures in children with supracondylar fractures of the humerus. J Pediatr Orthop..

[3] Reigstad O, Thorkildsen R, Grimsgaard C, Reigstad A, Røkkum M (2011). Supracondylar fracture with circulatory failure after reduction, pinning, and entrapment of the brachial artery: excellent results more than 1 year open exploration and revascularization. J Orthop Trauma..

[4] Omid R, Coi PD, Skaggs DL (2008). Supracondylar humeral fractures in children. J Bone Joint Surg Am..

[5] Robb JE (2009). The pink, pulseless hand after supracondylar fracture of the humerus in children. J Bone Joint Surg Br..

[6] Luria S, Sucar A, Eylon S, Pinchas-Mizrachi R, Berlatzky Y, Anner H, Liebergall M, Porat S (2007). Vascular complications of supracondylar humeral fracture in children. J Pediatr Orthop B..

[7] Omid R, Choi PD, Skaggs DL (2008). Supracondylar Supracondylar humeral fractures in children. J Bone Joint Surg Am..

[8] Babal JC, Mehlman CT, Klein G (2010). Nerve injuries associated withp supracondylar humeral fractures: a meta-analysis. J Pediatr Orthop..

